# Aneurism

**Published:** 1905-12-09

**Authors:** 


### Progress in Medicine and Surgery.

### ANEURISM.

**On the Surgical Treatment of Aneurisms.**—At the present time operations for the relief and cure of aneurism are made more safe and efficacious in proportion to the degree of asepsis that is obtainable, so that the great dread of secondary hæmorrhage is almost a thing of the past, and surgeons are more and more inclined to attack the affected artery either at or in close proximity to, the aneurism itself. In some cases, however, the tumour is so placed as to be either quite out of reach or its proximal artery is inaccessible. In these cases the distal or the distant proximal ligature have still to be employed. Dunn [1] describes such a case when he relates that of a man with an innominate aneurism. The patient was a man of forty with a history of syphilis eighteen years previously. A well-marked pulsating swelling was present beneath the origin of the right sterno-mastoid and he had neuralgic pains shooting up the right side of the head and into the right shoulder joint. The right common carotid was tied above the omo-hyoid and the subclavian below the clavicle, the latter being made possible by raising the shoulder. He made a good recovery and three months later was able to do his work. The sac of the aneurism had shrunk considerably, although some expansile pulsation could still be felt in it. The pain was cured but slight weakness of the right arm and immobility of the right vocal cord remained. Another most interesting recent case of intra-cranial aneurism was under the care of Berry.[2] A woman of forty-five had sudden violent pain over the right orbit twelve hours after a confinement. The pain was followed by the sensation of something snapping and was accompanied by a loud rushing sound. Three weeks later she became insane and had proptosis and ptosis of the right eye. Five years later, after regaining her sanity, she had severe right-sided headache, a rushing noise in the head which became less on pressing on the right carotid, impaired vision, and loss of sensation and motion in the right eye, and anæsthesia of the side of the face and vertex. Over the right temple a distinct but distant systolic bruit was heard. A diagnosis of aneurism of the internal carotid in the region of the cavernous sinus was made and the right internal carotid was tied one inch above the bifurcation. The rushing noise and the bruit disappeared, the pain was much relieved, and the proptosis lessened. She died from interstitial nephritis nearly a year later. Ligature of the innominate artery itself has only been performed thirty-six times, and of these only eight recovered from the operation. And it says a great deal for the results of aseptic methods that whereas the mortality of the whole series is about 75 per cent. that of the cases occurring since 1871 is only 25 per cent. The latest recorded case is by Sheen,[3] the patient was a labourer aged forty-six. He had an aneurism of the second and third parts of the right subclavian, the symptoms of which began six months previous to admission. The innominate and the right common carotid arteries were tied at the same time, both being reached by a median incision. Pulsation and pain returned almost immediately and it was thought that the ligature of the innominate must have loosened at the time of tying. Two months later a second attempt was made to tie the innominate, but success was prevented by alarming venous bleeding. A month after, the second part of the subclavian was tied after cutting the outer part of the scalenus anticus. The ligature was tied close to the aneurism. The aneurism consolidated and the man was well eight and a half months after the first operation. Sheen adds to the account of his case an abstract of all previously recorded cases, together with some comments. More than half the fatal cases died from secondary hæmorrhage, which set in at periods varying from the third to the thirtieth day after the operation. Sepsis alone accounted for death in three cases, and cerebral lesions in three more. As regards the method of ligature, the author regards silk as the best material, and favours the tight drawing of the thread so as to rupture the inner coats. The best method of preventing the ligature being untied by the blood-stream is to place two ligatures at a slight distance from one another and to have the proximal one held tight whilst the distal is being tied. If there is not room for this, Ballance's "stay" knot is the safest to use. But most desperate of all are cases of aneurism of the aorta in which an attempt is made to produce coagulation by introducing wire with or without subsequent electrolysis. Griffiths [4] describes such a case. A man of thirty-seven had severe pain and a pulsating swelling in the upper part of the abdomen. On opening the abdomen the tumour proved to be an aneurism of the upper part of the abdominal aorta which presented above the lesser curvature of the stomach. About six feet of fine silver wire was then pushed into the sac through a vulcanite canula and a current of twenty-five milliampères passed for fifteen minutes. The aneurism was felt to harden and the pulsation almost ceased. The patient died about five hours later and a post-mortem revealed that a loop of the wire had ascended into the thoracic aorta and clotting had occurred there. Recently Matas,[5] in America, has introduced a method of operation upon aneurisms which involves quite a new principle. He treats the coats of the artery as one would do the coats of intestine in performing any plastic operation. That is to say he opens the aneurism after the circulation has been controlled by a tourniquet, and having found the openings of the artery and its branches, proceeds to sew them up by stitches resembling the Lembert suture of intestinal work. After cutting away the redundant walls of the aneurism the remaining part is plicated and sewn together to form a solid cord. It seems difficult to appreciate the advantage of this complicated proceeding over the earliest of all operations for aneurism—namely, that of Anayllus who simply cut open the blood sac and tied the bleeding vessels. And there is no doubt that with modern asepis this method might often be employed in aneurisms of the limbs. The above plan applies to the case of a sacculated aneurism, and several successful cases of its application have been reported from America. Gibbon [6] describes one in which he so dealt with a large popliteal aneurism. Unfortunately the wound suppurated and a large quantity of pus had to be evacuated, but, strange to say, there was no secondary hæmorrhage. In sacculated aneurisms in which only a single opening exists between the sac and the artery from which it springs, Matas simply sews up this opening by interrupted sutures of chromic gut and the lumen of the artery is left and the main blood-stream is not interfered with. In such a case—the conditions for which must be very rare—the advantage of leaving the main arterial channel unblocked is obvious. But, on the other hand, there must be a grave risk of leaving a weak spot in the arterial wall which might lead to the recurrence of the aneurism.

[1] Lancet, June 3, 1905. [2] Ibid., July 22, 1905. [3] Annals of Surg., July, 1905. [4] Lancet, Aug. 12, 1905. [5] Annals of Surg., Feb., 1903. [6] Ibid., July, 1905.

